# Mitigating healthcare staffing shortages: Should healthcare workers with severe acute respiratory coronavirus virus 2 (SARS-CoV-2) household exposures work?

**DOI:** 10.1017/ice.2022.195

**Published:** 2023-07

**Authors:** Ana C. Blanchard, Valérie Lamarre, Josée Lamarche, Nathalie Audy, Caroline Quach

**Affiliations:** 1 Division of Pediatric Infectious Diseases, CHU Sainte-Justine, Montreal, Québec, Canada; 2 Department of Pediatrics, Faculty of Medicine, Université de Montréal, Québec, Canada; 3 Department of Microbiology, Infectious Diseases, and Immunology, Faculty of Medicine, Université de Montréal, Québec, Canada; 4 Infection Prevention and Control, CHU Sainte-Justine, Montreal, Québec, Canada; 5 COVID-19 Unit, Occupational Health and Services, CHU Sainte-Justine, Montreal, Québec, Canada; 6 Nursing Call Center, Network Activity Coordination Center, Nursing Care Division, CHU Sainte-Justine, Montreal, Québec, Canada; 7 Clinical Department of Laboratory Medicine, CHU Sainte-Justine, Montreal, Québec, Canada

## Abstract

In a tertiary-care, pediatric healthcare center in Québec, Canada, healthcare workers who reported a household exposure to confirmed coronavirus disease 2019 (COVID-19) cases were allowed to work. On repeated testing, 15% became severe acute respiratory coronavirus virus 2 (SARS-CoV-2)–positive by reverse-transcription polymerase chain reaction (RT-PCR), with no nosocomial transmission. Being asymptomatic and receiving a booster dose >7 days prior to exposure was protective against becoming SARS-CoV-2–positive by PCR.

In the province of Québec, Canada, the severe acute respiratory coronavirus virus 2 (SARS-CoV-2) omicron (BA.1) variant of concern (VOC) became predominant on December 20, 2021.^1^ Due to increased community transmission, healthcare workers (HCWs) increasingly reported significant exposures to confirmed household cases. On the basis of data suggesting that testing may reduce quarantine duration, previous provincial recommendations allowed HCWs to work despite contact with a confirmed SARS-CoV-2 infected household member if HCWs (1) had been vaccinated with 2 doses of coronavirus disease 2019 (COVID-19) vaccine >7 days before contact and (2) were asymptomatic.^2,3^ Given reduced 2-dose vaccine effectiveness against the SARS-CoV-2 omicron variant,^4^ new provincial guidelines^2^ recommended quarantine of HCWs exposed to confirmed household cases, regardless of symptoms. However, due to significant staffing shortages at that time, we allowed HCWs to work after household exposures if they were asymptomatic^5^ or mildly symptomatic. These HCWs had to have received ≥2 doses of vaccine, follow exemplary measures (ie, wear a procedure masks or an N95 respirator, eat alone in a closed room, monitor symptoms), and have reverse-transcription polymerase chain reaction (RT-PCR) testing for SARS-CoV-2 every 3 days until 7 days after the end of the contagious period of the household case. Here, we describe the risk for HCWs who reported a household contact who became SARS-CoV-2–positive by RT-PCR. We also evaluated the risk of nosocomial transmission and outbreaks.

## Methods

### Study design

In this retrospective cohort study, we identified HCWs who reported a household contact with a PCR-confirmed COVID-19 case to the Occupational Health and Safety (OHS) starting December 20, 2021.

### Setting and participants

CHU Sainte-Justine is a tertiary-care hospital located in Montreal, Canada. At the beginning of the COVID-19 pandemic, OHS established a call center, staffed 24 hours per day, 7 days per week with nurses who evaluated all HCWs with symptoms or exposures, under the supervision of human resources staff and the medical direction of the infection prevention and control (IPAC) team. A testing clinic is also available on-site for RT-PCR testing, which is done in our diagnostic microbiology laboratory.^6^

### Variables and measurement

The main outcome was a RT-PCR test positive for SARS-CoV-2. Collected variables included vaccine doses and dates, RT-PCR results and dates, self-reported symptoms at initial test, cases of nosocomial COVID-19 cases in patients (symptom onset ≥3 days after admission), and outbreaks among HCWs (≥2 cases in a same sector). No demographic data were collected. Outbreaks and nosocomial cases were identified through daily analysis of COVID-19 data reported to the Quebec Ministry of Health and Social Services as part of the usual IPAC surveillance process.

### Statistical analysis

The incidence of RT-PCR positivity for SARS-CoV-2 in household-exposed HCWs was assessed using descriptive statistics. A Kaplan-Meier curve and a logistic regression were used to assess the association of having symptoms (primary exposure) with the risk of SARS positivity by RT-PCR, adjusted for third-dose vaccination status (valid if >7 days). All analyses were performed using Stata version 17.0 software (StataCorp, College Station, TX).

### Ethical consideration

Because this was a process evaluation using data collected through our usual process of care, we obtained a waiver from the research ethics committee.

## Results

**Fig. 1.**
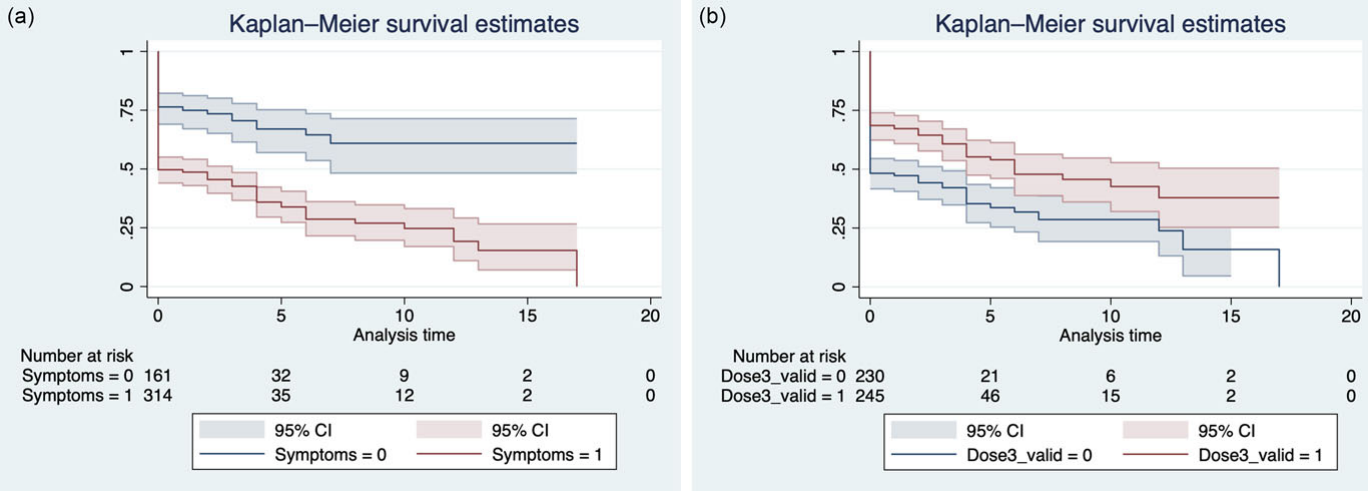
Survival curves for remaining RT-PCR negative (a) stratified by presence of symptoms at first test and (b) stratified by vaccination status.

From December 20, 2021, to January 17, 2022, 475 HCWs reported a household contact with an RT-PCR–confirmed case of COVID-19. Overall, 237 HCWs (49.9%) remained SARS-CoV-2–negative by RT-PCR. Of those who became positive on RT-PCR, 196 (82.4%) of 238 were positive on their initial test. The others became SARS-CoV-2-positive a median of 4 days afterward (IQR, 3–6). Initially asymptomatic exposed HCWs were 3 times more likely to remain SARS-CoV-2–negative by PCR compared with those who had symptoms (odds ratio [OR], 3.78; 95% CI, 2.50–5.73), when adjusted for having received a third vaccine dose >7 days prior to exposure (Table 1). Figure 1 illustrates the time to event of remaining SARS-CoV-2–negative stratified on the presence of symptoms and a third vaccine dose.

**Table 1.** Characteristics of Exposed HCWs and Odds of Remaining SARS-CoV-2–Negative by RT-PCR

Variable	RT-PCR		Total, No. (%)	Crude Odds Ratio of Remaining SARS-CoV-2– Negative by RT-PCR (95% CI)	Adjusted Odds Ratio of Remaining SARS-CoV-2–Negative by RT-PCR (95% CI)
	Negative	Positive			
Absence of symptoms at initial test	115	46	161 (33.9)	3.93 (2.61–5.93)	3.78 (2.50–5.73)
Third vaccine dose valid	143	102	245 (51.6)	2.02 (1.41–2.92)	1.88 (1.29–2.77)
Initial RT-PCR positive	…	196	196 (41.2)	…	…
Total	237	238	475		

Note. RT-PCR, reverse transcription polymerase chain reaction; CI, confidence interval.

During that period, 10 outbreaks occurred among HCWs, with a median of 3 HCWs per outbreak (IQR, 3–6). None were associated with a HCW who reported a household contact, although some were associated with a HCW who had an unknown household contact. which was identified during the investigation done following the positive HCW’s RT-PCR result. Overall, 9 nosocomial COVID-19 cases were identified in patients. In 3 cases, an epidemiological link with an infected parent or a visitor was clear. Also, 3 patients were exposed to SARS-CoV-2–positive patients in the hematology-oncology day center. For the remaining 3 patients, no source was identified. The list of HCWs who had cared for these 3 patients in the 7 days prior to infection was carefully cross tabulated with HCWs who had reported a household contact and became positive. None was identified as the source.

## Discussion

We summarized our experience allowing HCWs who reported a significant household exposure to a confirmed COVID-19 case to work if they had received ≥2 doses of vaccine and a negative initial PCR result.

Recent studies have reported that household secondary attack rates (SARs) were 35.8% in 2021,^7^ that the SARS-CoV-2 omicron variant had a higher SAR than the SARS-CoV-2 delta variant in a recent study, and that boosted individuals have an SAR of 25% for the SARS-CoV-2 omicron variant.^8^ We report an SAR of 50% overall, which decreased to 42% among those who had received booster vaccinations. Our SAR was higher than the SAR in the Danish study, possibly because the outcomes assessment was more complete and used RT-PCR, whereas secondary cases in the Danish study may have been assessed using a rapid antigen detection test, which has lower sensitivity.^6^ Moreover, a large proportion of our HCWs were SARS-CoV-2–positive by RT-PCR upon assessment, indicating that they may not have been a household contact but rather the index case or may have been infected at the same time as their household member through a common source.

HCWs who were SARS-CoV-2–positive by RT-PCR upon initial evaluation were isolated. Of the remaining 279 initially SARS-CoV-2–negative HCWs who worked, 42 (15%) became positive. The presence of symptoms at initial testing following household exposure was associated with an increased risk of a positive RT-PCR. Adjusting for the presence of symptoms, having a valid third vaccine dose increased the odds of remaining SARS-CoV-2 negative by 88%. IPAC measures in place mitigated the risk of transmission to patients, even if symptomatic exposed HCWs were allowed to work. These findings suggest that prioritizing asymptomatic, fully vaccinated HCWs to continue working despite a known household exposure is likely safe.

Our study had several limitations. It was not a research study but rather an OHS and IPAC initiative to reduce staffing shortages for patient safety. Demographic, socioeconomic factors and the degree of household exposure (ie, number of infected contacts, duration of contact and presence of symptoms of the household index case) were not systematically collected by OHS. The data were based on self-reported household contacts, whether HCWs had symptoms or not. Although HCWs may not have reported all known exposures, they needed to be assessed by OHS to obtain paid leave. We expect that the data are complete. In this real-life setting, it was impossible to include all known and unknown household index cases. Our objective was not to document the household SAR but rather to evaluate whether it was safe to allow initially SARS-CoV-2–negative HCWs with known household exposures to work. The findings of this study are limited by the absence of a control group (ie, HCWs without household exposures).

In a pediatric hospital where vaccination rates among HCWs are high, the risk of staffing shortages has nonnegligible effects on quality of care and patient safety. Our data support the assertion that allowing fully vaccinated asymptomatic HCWs who have reported a household contact with a confirmed COVID-19 case to work is likely safe in the context of exemplary infection prevention measures and repeated testing when staff shortage is anticipated.
